# Bilateral Diffuse Uveal Melanocytic Proliferation Presenting as Small Choroidal Melanoma

**DOI:** 10.1155/2011/740640

**Published:** 2011-12-20

**Authors:** J. N. Ulrich, S. Garg, G. K. Escaravage, T. M. Meredith

**Affiliations:** Department of Ophthalmology, University of North Carolina at Chapel Hill, CB 7040, Chapel Hill, NC 27599, USA

## Abstract

*Purpose*. To describe a patient with Bilateral Diffuse Uveal Proliferation who presented initially with a clinical picture consistent with choroidal melanoma. *Methods*. Presentation of a clinical case with fundus photos, fluorescein angiography, and optical coherence tomography. *Results*. A 70-year-old Caucasian male with history of esophageal cancer presented with an asymptomatic pigmented choroidal lesion in his left eye initially diagnosed as choroidal nevus. This lesion enlarged over the course of a year and developed orange pigment and increased thickness. A metastatic workup was negative, and a radioactive iodine plaque was placed on the left eye. Over the next six months, the visual acuity in his left eye decreased. His clinical picture was consistent with unilateral Diffuse Uveal Proliferation. A recurrence of his esophageal carcinoma with metastasis was discovered and palliative chemotherapy was initiated. Although his visual acuity improved in the left eye, similar pigmentary changes developed in the right fundus. His visual acuity in both eyes gradually decreased to 20/200 until his death a year later. *Conclusion*. BDUMP should always be considered in the differential diagnosis of patients with pigmented fundus lesions and a history of nonocular tumors.

## 1. Introduction

Bilateral Diffuse Uveal Melanocytic Proliferation (BDUMP) was first described by Machemer in 1966 [1]. It is thought to be a paraneoplastic condition caused by diffuse proliferation of benign melanocytes in the outer choroid which are histopathologically unrelated to the primary nonocular tumor. The typical clinical findings are nevi-like, multifocal reddish patches at the level of the retinal pigment epithelium (RPE) in the posterior pole which hyperfluoresce on fluorescein angiography [2, 3].

We report an atypical intial presentation of BDUMP mimicking a small choroidal melanoma. 

## 2. Case Report

**Figure 1:**
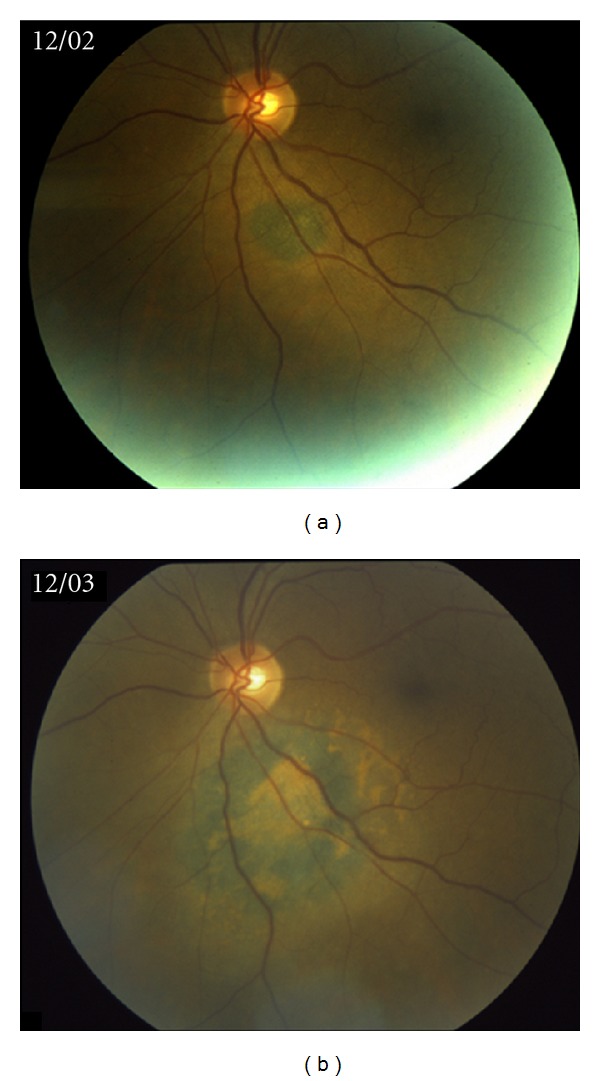
Evolution of choroidal lesion, left eye, over the course of one year.

**Figure 2:**
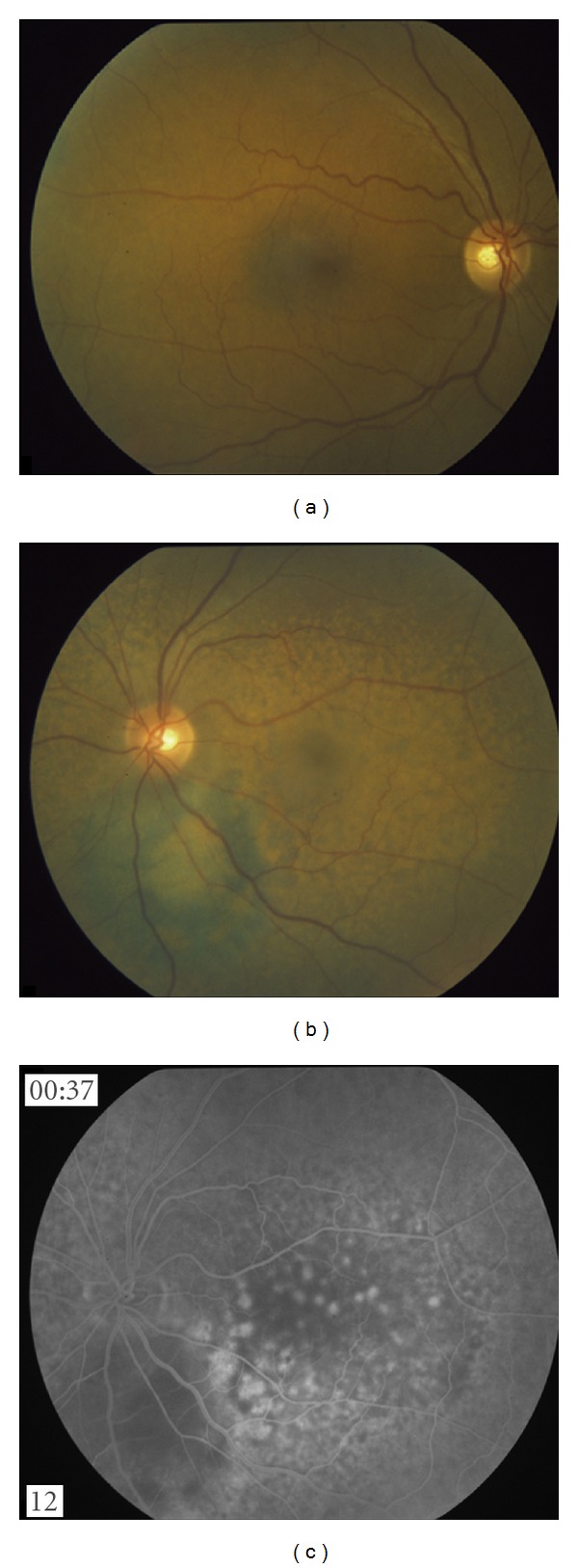
The right fundus is unremarkable. Left eye, six months after radioactive iodine treatment: fine, patchy, pigmentary changes throughout the posterior pole with an unchanged appearance of the original pigmented lesion. Fluorescein angiography shows blocking of the original lesion and fine hyperfluorescence corresponding to the areas with pigmentary change on fundus photos.

**Figure 3:**
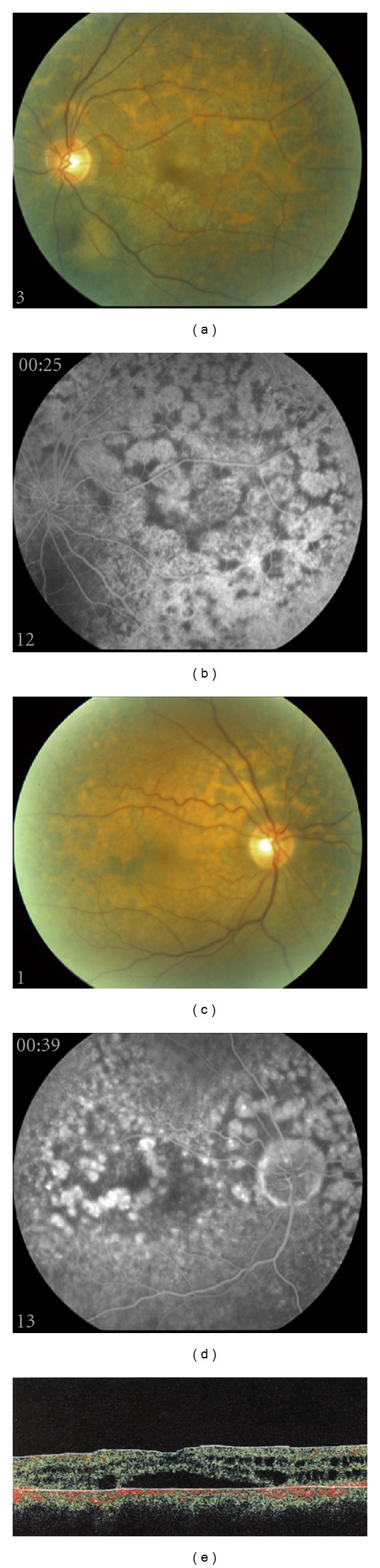
Color fundus photos of both eyes show nummular plaque-like patches at the level of the retinal pigment epithelium. Fluorescein angiography shows round areas of transmission defects as well as fine staining within these regions. OCT of the left eye shows intraretinal cysts, subretinal fluid, as well as disruption of the retinal pigment epithelium.

A 70-year-old Caucasian male with a history of esophageal cancer status after surgery, chemotherapy, and radiation presented with a 2 × 2 mm flat pigmented choroidal lesion inferior to the optic nerve in his left eye (OS) on a routine eye examination. This lesion enlarged over the following 12 months to approximately 5 × 5 mm with 1 mm elevation and a significant amount of lipofuscin (Figure 1). After normal systemic workup including CT and PET scan treatment with a Radioactive Iodine (RAI) plaque OS was performed. Over the next 6 months, the patient developed progressive vision loss from 20/20 to 20/400 OS. On fundus examination, multiple small patches of pigment alteration at the level of the RPE developed in the posterior fundus OS. Fluorescein angiography showed blocking of the original lesion as well as diffuse, stippled hyperfluorescence in the areas of pigment alteration (Figure 2). During this time, a recurrence of his esophageal cancer with widespread metastases was detected, and he underwent a cycle of palliative chemotherapy. His vision OS temporarily improved to 20/40 with resolution of subretinal fluid. The right eye developed similar findings of nummular plaque-like patches on the level of the RPE (Figure 3). Fluorescein angiography showed circular areas of transmission defects as well as fine staining within these regions (Figure 2). Optical coherence tomography (OCT) demonstrated subfoveal fluid as well as disruptions of the retinal pigment epithelium, corresponding to the plaque-like areas on the fundus photographs and the areas of staining on fluorescein angiography. The subretinal fluid was nonresponsive to topical and periocular steroids but diminished with treatment of oral prednisone. However, despite resolution of subretinal fluid, his visual acuity gradually deteriorated to 20/200 in both eyes until his death one year later.

## 3. Discussion

BDUMP is a rare paraneoplastic condition with approximately 30 reported cases in the literature. Patients typically present with sudden bilateral vision loss which antedates the discovery of a primary nonocular tumor in approximately half of the cases. Survival after diagnosis is between 8 and 24 months [2]. Gass et al. described the findings in BDUMP as thickened choroid with both hypo- and hyperpigmentation and nevi-like structures mainly in the posterior pole as well as annular patches of RPE alterations [2]. More recently, Wu et al. reported a variety of BDUMP with round patches of RPE loss as the predominant finding (cancer-associated nummular loss of pigment epithelium) [4] without choroidal thickening or choroidal pigmented lesions.

The initial presentation of our patient was unique. The patient had an asymptomatic unilateral small pigmented lesion which was initially diagnosed as a choroidal nevus. The lesion enlarged over the course of one year and demonstrated classic signs of a small choroidal melanoma (lipofuscin, elevation, low internal reflectivity). At that time, there were no signs of any fundus abnormalities in the fellow eye. Consultation with two ocular oncology experts was arranged who agreed with the diagnosis of melanoma and subsequent treatment with RAI plaque placement. Typical signs of BDUMP did not develop until months after the plaque treatment. It was then that a recurrence of his primary esophageal tumor was detected as well. Of note, the original elevated choroidal lesion remained hypofluorescent on fluorescein angiography whereas the subsequent lesions that developed were flat, smaller and underwent a transformation on fluorescein angiography from pinpoint hyperfluorescence to larger window defects with areas of staining later in the course.

This unique case illustrates that BDUMP should always be considered in the differential diagnosis of patients with pigmented fundus lesions and a history of nonocular tumors even if a systemic workup reveals no signs of recurrence upon presentation.
